# Prolonged Mitotane Administration in Metastatic Adrenocortical Carcinoma With Over a Decade of Survival: A Case Series

**DOI:** 10.1155/crie/9096041

**Published:** 2025-11-25

**Authors:** Lorenzo Tucci, Mengjie Xu, Miriam Asia, Kassiani Skordilis, Maninder S. Kalkat, Madava Djearaman, Robert P. Sutcliffe, Yasir S. Elhassan, Alessandro Prete, Cristina L. Ronchi

**Affiliations:** ^1^Department of Metabolism and Systems Science, College of Medicine and Health, University of Birmingham, Birmingham, UK; ^2^Department of Medical and Surgical Sciences Department, Alma Mater University of Bologna, Bologna, Italy; ^3^Division of Endocrinology and Prevention and Care of Diabetes, IRCCS Azienda Ospedaliero-Universitaria di Bologna, Bologna, Italy; ^4^Department of Endocrinology, Taihe Hospital, Hubei University of Medicine, Shiyan, China; ^5^Department of Endocrinology, Queen Elizabeth Hospital Birmingham NHS Trust, Birmingham, UK; ^6^Department of Cellular Pathology, Queen Elizabeth Hospital Birmingham NHS Trust, Birmingham, UK; ^7^Department of Thoracic Surgery, Queen Elizabeth Hospital Birmingham NHS Trust, Birmingham, UK; ^8^Department of Imaging, Queen Elizabeth Hospital Birmingham NHS Trust, Birmingham, UK; ^9^Department of Hepatobiliary Surgery, Queen Elizabeth Hospital Birmingham NHS Trust, Birmingham, UK; ^10^NIHR Birmingham Biomedical Research Centre, University of Birmingham and University Hospitals Birmingham NHS Trust, Birmingham, UK

**Keywords:** adrenocortical cancer, local therapy, long-term survival, low tumour burden, metastases, minimally invasive treatment, mitotane, recurrences

## Abstract

**Background:**

Reports of long-survival patients (>10 years) with metastatic adrenocortical carcinoma (ACC) and prolonged treatment with mitotane alone (>5 years) are rare. We present four patients treated for low tumour burden (LTB) metastatic ACC using multidisciplinary approaches, including long-term mitotane, who maintained a relatively good life quality for over 10-year survival.

**Case Series Presentation:**

Four patients (one female) aged 34–52 years underwent adrenalectomy for ACC between 2004 and 2013. Two patients presented only with flank pain, while two presented with overt Cushing's Syndrome. Ki-67 index ranged from <1% to 37%, and European Network for Study of Adrenal Tumour (ENSAT) tumour stage ranged from 2 to 3. Time to first recurrence ranged from 8 to 48 months. Overtime, all patients experienced recurrent metastatic disease, mostly in lungs or liver. From 2008 to 2024, we recorded a total of two stereotactic ablative radiotherapies, one microwave and eight radiofrequencies ablations, 10 liver and eight lung resections and three selective excisions for other abdominal metastases. Mitotane treatment lasted from 7.7 to 12.9 years, with mitotane average dose and plasma levels equal to 2.6 ± 0.9 g/day (±standard deviation) and 20.4 ± 5.3 mg/L, respectively. Our patients developed different degrees of mitotane-induced hypothyroidism, hypogonadism, hypercholesterolaemia and mineralocorticoid insufficiency as well as episodes of neurologic and gastrointestinal side effects, which were countered with continuous dose adjustments, hormonal replacement therapies and specific treatments. Specialist nurses provided continuous support for mitotane dose titration and management of mitotane-induced adverse effects. All the patients were still taking mitotane at last available follow-up visit without radiological evidence of tumour manifestations, with a follow up duration ranging from 11.5 to 20 years from initial surgery.

**Conclusion:**

Metastatic ACC should be managed by multidisciplinary tailored approaches, including close surveillance and local therapies (LT). In patients with LTB, prolonged mitotane therapy can represent a safe option to slow down disease progression and contribute to prolonging survival.

## 1. Background

Adrenocortical carcinoma (ACC) is a rare malignancy (0.5–2 cases/million/year) with an overall 5-year survival rate equal to 65%–82%, 56%–68%, 41%–55% and 10%–20% for tumour stages according to the European Network for Study of Adrenal Tumour (ENSAT) 1, 2, 3 and 4, respectively, and a median life expectancy of 15 months at Stage 4, with metastases frequently localised in the liver, lungs and bones [1–6]. The only drug approved for ACC by the European Medical Agency and by the Food and Drug Administration is mitotane, an adrenolytic agent with a long half-life, a narrow therapeutic window and numerous and potentially severe side effects. Therefore, it is recommended that patients taking mitotane undergo close clinical and laboratory monitoring [7]. International guidelines recommend using mitotane either in an adjuvant setting for patients who underwent complete surgical resection but are considered at high risk for recurrence or in patients with advanced ACC aiming to increase overall and progression-free survival (PFS) [2, 3, 8, 9]. However, due to lack of evidence, current guidelines do not provide clear recommendations about the benefits and safety of mitotane treatment for periods longer than 5 years [2, 3, 8, 9]. In case of disease progression, guidelines suggest to associate mitotane with a systemic chemotherapy based on a combination of etoposide, cisplatin and doxorubicin (EDP), but side effects are common and the response rate remains unsatisfactory, that is, 15%–35% of treated cases [3, 8]. Guidelines also recommend to use local therapies (LT), especially in patients with low tumour burden (LTB, lesions smaller than 5 cm in size associated with a disease-free interval greater than 9 months), as additional options for the management of metastatic ACC, independently from systemic therapy [2]. LTs may include radiofrequency ablation (RFA), transcatheter arterial chemoembolization and microwave ablation, all of which have been proved to have a positive impact in controlling localised metastatic lesions, mostly in patients with LTB in the liver and lungs [9–11]. Recent studies also investigated stereotactic ablative radiotherapy (SABR) for metastatic ACC with promising results, albeit heterogeneous [11, 12]. Potential benefits of LTs and resurgeries in metastatic ACC can be found even in articles from the early '90s, especially in those about long-term survivors (> 10 years) with metastatic ACC [13–23]. However, in these articles, patients had been treated with different approaches, and no or scarce descriptions of systemic therapies are provided [13–23]. Moreover, even if some patients surpassed 20 years of survival, there is no exhaustive description of prolonged use (> 5 years) of mitotane in monotherapy [13–23].

**Figure 1:**
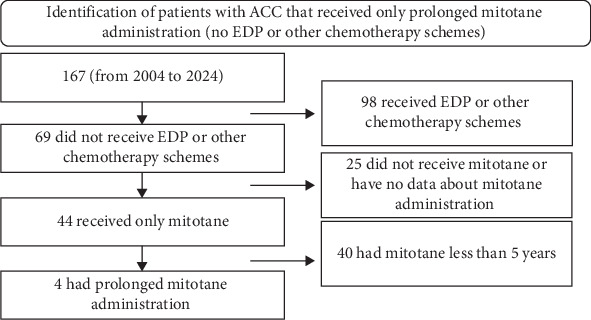
Identification of patients with adrenocortical cancer that received only prolonged mitotane administration (no etoposide–doxorubicin–cisplatin or other chemotherapy schemes). ACC, adrenocortical cancer; EDP, etoposide–doxorubicin–cisplatin.

Hence, nowadays it is not clear whether long-term treatment with mitotane could be useful and safe for patients with recurrent LTB metastatic ACC not suitable for or not willing to undergo EDP. In the attempt to help filling this void, we checked our records looking for patients treated with prolonged mitotane administration without EDP or other chemotherapy schemes (Figure 1). Therefore, we present four cases of long-term metastatic ACC survivors, which had recurrent LTB and were managed by our multidisciplinary Team (MDT) through a combination of multiple surgical resections and LTs, as well as long-term mitotane therapy for over 10 years. All cases are reported following the CARE guidelines [24].

## 2. Case 1

### 2.1. Case Presentation

**Figure 2:**
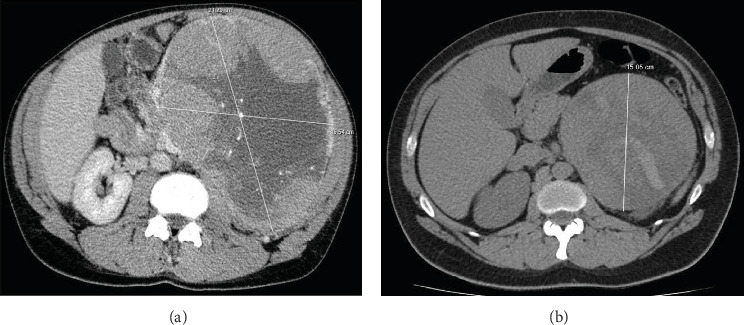
Computerised tomography at diagnosis of Case 1 (a) and Case 3 (b). (a) Computerised tomography of adrenocortical cancer at diagnosis time for Case 1; (b) computerised tomography of adrenocortical cancer at diagnosis time for Case 3.

In September 2009, a 49-year-old man with unremarkable past medical history, underwent a thorax–abdomen–pelvis (TAP) contrast-enhanced computerised tomography (CT) scan for abdominal pain, which revealed a left upper quadrant mass measuring 21 cm × 19 cm × 16 cm with diffuse calcifications and central necrosis with no evidence of metastatic disease (Figure 2a). Details of case presentation at diagnosis are in Table 1. The patient underwent excision of the 3.8 kg left retroperitoneal mass in October 2009. The histological examination showed a diagnosis of conventional ACC with Weiss score 5/9, ENSAT Stage 3 (invasion of surrounding tissue), Resection status unknown (Rx), and Ki-67 index <1%. We retrospectively calculated the S-GRAS score as 3. S-GRAS score is based on five clinical/histopathological variables (i.e., age, symptoms at diagnosis, ENSAT tumour stage, resection status and Ki-67 index) as previously published [25].

### 2.2. Long-Term Pharmacological Treatment With Mitotane

The patient was referred to our department in March 2011, after the first disease recurrence (see paragraph below), when the case was reviewed by our adrenal MDT. Mitotane was started according to a high-dose saturation regimen alongside hydrocortisone replacement therapy [26]. The patient has been on continuous mitotane treatment from March 2011 to June 2024 (time of last record) but was briefly discontinued in 2013 due to adrenal crisis, with a Time in Target Range (TTR) of 41.7% (Table 1) [27]. The patient experienced one severe episode of neurotoxic side effects (slurred speech in April 2012) and developed mitotane-induced hypogonadism (June 2012), mineralocorticoid insufficiency (January 2013) and hypothyroidism (December 2015). Mitotane dose was temporary reduced to face neurotoxicity, while hypogonadism was treated with intramuscular nandrolone in the first place, then dihydrotestosterone skin gel, and fludrocortisone and levothyroxine were used for mineralocorticoid insufficiency and hypothyroidism, respectively. The patient was well compliant to all medications throughout the follow-up period and reported both a good tolerability of mitotane after treatment of related side effects and a relatively good quality of life.

### 2.3. Long-Term Radiological Surveillance and Additional Treatments

Starting from June 2010, the patient underwent a TAP contrast-enhanced CT scan every 3–6 months. The patient had his first recurrence in December 2010, which presented with millimetric bilateral lung nodules, also confirmed through 18-fluorodeoxyglucose positron emission tomography-CT (FDG-PET-CT). In ~15 years of follow up, the patient had nine lung recurrences (min–max size: 6–14 mm) treated with two bilateral VAT lung excisions (2010 and 2011), six RFAs (2016, 2017, 2018, two in 2020, 2023) and one microwave ablation (2021, too close to the heart to perform RFA), a 5 cm recurrence in the 8th right rib treated with SABR (2017) and a 3 cm colon metastasis removed with polyp excision through colonoscopy (2017). The patient remained disease-free at the last surveillance CT scan in September 2024.

## 3. Case 2

### 3.1. Case Presentation

In November 2011, a 52-year-old female was diagnosed adrenocorticotropic hormone independent Cushing's Syndrome with androgen excess and associated with a 22 cm left adrenal mass (Table 1). Her past medical history included hysterectomy with bilateral oophorectomy for metrorrhagia (2006) and asthma. Her medical therapy included oestrogen replacement therapy (stopped in October 2011), ciclesonide and salbutamol inhalers. The patient underwent open left adrenalectomy and nephrectomy in January 2012, and the histology report was consistent with conventional ACC, Weiss score 9/9, ENSAT Stage 2, R0, Ki-67 index 37% and S-GRAS score 5. At discharge, glucocorticoid replacement therapy was prescribed, and the patient was referred to our department. Physical examination findings were available only from postoperative assessment and revealed overweight (body mass index 27.87 kg/m^2^) and no signs of androgen excess.

### 3.2. Long-Term Pharmacological Treatment With Mitotane

After MDT evaluation, adjuvant mitotane was started in June 2012 according to the high-dose saturation regimen alongside hydrocortisone replacement therapy [26]. The patient has been on continuous mitotane treatment from June 2012 to July 2024 (time of last record) but was discontinued for 4 weeks between February and March 2023 for surgical removal of liver metastases and for 2 weeks in July 2024 for markedly high mitotane plasma levels (41.4 mg/L). Overall, TTR was 65.4% (Table 1) [27]. Overtime, the patient experienced two severe episodes of neurotoxic and gastrointestinal side effects (diarrhoea, indigestion and slurred speech in August 2012, brain fog in October 2012) and developed mitotane-induced hypothyroidism (January 2017). Mitotane dose was temporary reduced for both toxicity events, with also temporary use of ondansetron for gastrointestinal (nausea and vomiting) adverse effects. The patient had outstanding compliance with all her medications, including mitotane, which was overall well tolerated after side effect treatments.

### 3.3. Long-Term Radiological Surveillance and Additional Treatments

Starting from April 2012, the patient underwent a TAP contrast-enhanced CT scan every 3–6 months, alongside with a liver-specific abdomen magnetic resonance imaging (LS-AMRI) from September 2015, when she presented her first disease recurrence in the liver, which also was demonstrated to be FDG avid at PET-CT. In 12 years of follow-up, the patient had 14 liver recurrences (min–max size: 5–35 mm), for which she had three non-anatomical resections (NAR) of the liver (2015, 2019, 2022), one hemi-hepatectomy (2017) and one SABR (2022), and four lung metastases (min–max size: 5–9 mm) for which she had one RFA (2021), two VATs (both in 2016) and two wedge lung excisions (2018, 2024). The patient remained disease-free at the last surveillance CT scan in January 2025.

## 4. Case 3

### 4.1. Case Presentation

In May 2013, a 34-year-old male was diagnosed with adrenocorticotropic hormone-independent Cushing's Syndrome with androgen excess and associated with a 15 cm left adrenal mass (Figure 2b), which was causing abdominal pain (Table 1). The patient had an unremarkable personal and familiar medical history. The patient underwent an open left adrenalectomy in September 2013, and hydrocortisone replacement therapy was started. The histology report was consistent with 21 cm × 16 cm × 10 cm conventional ACC, 2.2 kg, Weiss score 8/9, Ki-67 index 4.4%, R0, ENSAT Stage 3 (invasion of surrounding tissue) and S-GRAS score 2.

### 4.2. Long-Term Pharmacological Treatment With Mitotane

After MDT discussion, adjuvant mitotane was started in November 2013 according to the high-dose saturation regimen, but due to gastrointestinal side effects, the patient did not exceed 4500 mg daily [26]. The patient has been on continuous mitotane treatment from November 2013 to July 2024 (time of last record) but was discontinued for 2 weeks in September 2015 and June 2016 due to high mitotane plasma levels. In total, TTR was 41.7% (Table 1) [27]. In 11 years, the patient experienced fatigue with brain fog in December 2013, nausea with episodes of vomiting in January 2014 and developed mitotane-induced hypogonadism in August 2014 and hypothyroidism in August 2019. Mitotane dose was temporary reduced to face neurotoxicity and gastrointestinal (nausea and vomiting) side effects with temporary administration of ondansetron to treat the latter. Hypogonadism was treated with intramuscular nandrolone in the first place, then testosterone skin gel and levothyroxine was administrated to correct hypothyroidism. The patient had good compliance to all medications and reported a relatively good life quality after side effect treatments.

### 4.3. Long-Term Radiological Surveillance and Additional Treatments

Starting from November 2013, the patient underwent a TAP contrast-enhanced CT scan every 3–6 months, alongside with a LS-AMRI from August 2015, when the first liver recurrence was detected. From October 2015 to August 2024, the patient experienced 14 liver metastases (min–max size: 5–31 mm), treated with one hemi-hepatectomy (2017) and four NARs (2015, 2017 with hemi-hepatectomy, two in 2018), one RFA (2017), two retroperitoneal lymph node recurrences (24 and 21 mm) which were surgically removed (2022), and two lung metastases (7 and 9 mm) treated by VAT and lung wedge excision (2021 and 2024, respectively). The patient remained disease-free at the last surveillance CT scan in March 2025.

## 5. Case 4

Unfortunately, we were not able to retrieve Case 4 original documentation reporting exact dates of surgeries and sizes of primary ACC and metastases. Nevertheless, we consider this case to be useful for the purpose of the article.

### 5.1. Case Presentation

In 2004, a 39-year-old man with an unremarkable medical history was admitted due to right flank pain and underwent a TAP CT scan, which revealed a right adrenal mass. No clinical or biochemical evidence of hormonal excess was documented. He underwent a right adrenalectomy a few weeks after, and histology was consistent with conventional ACC (Weiss score unavailable), ENSAT Stage II, Rx, Ki-67 index 8%–10%, S-GRAS 3. The patient was referred to our department only in January 2017 for suspected second liver metastases, which were ruled out with LS-AMRI.

### 5.2. Long-Term Pharmacological Treatment With Mitotane

According to the ESE–ENSAT Clinical Practice Guidelines, adjuvant mitotane therapy is recommended in patients with a high-risk of recurrences or incomplete resection [2, 3]. As patients with previous disease recurrences are at higher risk of developing further relapses, mitotane was started in January 2017, for example, with a high-dose saturation regimen alongside hydrocortisone replacement therapy [6, 26]. The patient has been on continuous mitotane treatment from January 2017 to July 2024 (time of last record) without interruptions, with a TTR of 27.3% (Table 1) [27]. The patient developed drug-induced hypothyroidism in March 2017, as well as hypercholesterolaemia and hypogonadism in October 2018, and experienced adrenal crisis following mineralocorticoid insufficiency in July 2022, treated with levothyroxine, atorvastatin, testosterone gel and fludrocortisone, respectively. The patient adhered well to all prescribed medications and reported a satisfactory quality of life following the management of side effects.

### 5.3. Long-Term Radiological Surveillance and Additional Treatments

Starting from 2004, the patient underwent a CT scan every 3–6 months alongside with an LS-AMRI from December 2016. The patient had a first liver recurrence in 2008, treated with partial hepatectomy, and a local recurrence surgically treated, followed by radiotherapy to the adrenal bed (60 Gy in 30 fractions) in 2013. The patient remained disease-free at the last follow-up imaging in September 2024.

## 6. Continouos Monitoring by Adrenal Specialist Nurse

At our centre, we offer a nurse-led service for continuous clinical and biochemical assessment as well as treatment monitoring for patients with ACC. Hereby, patients' compliance and tolerance to mitotane were assessed monthly for the first 12 months, then quarterly, by an adrenal specialist nurse that kept track of subjective symptoms, physical exam findings, lab test results and plasma mitotane concentrations. In parallel, the nurse provided continuous support to encourage self-management of mitotane-related adrenal insufficiency, such as reinforcing sick-day rules and the use of emergency injection kits. All patients were initiated on hydrocortisone 50 mg/day (20-20-10 mg) concomitantly with mitotane, with subsequent dose adjustments made by the specialist nurse and/or the patients themselves, after appropriate training, according to intercurrent illness or stress-dosing requirements.

## 7. Discussion

**Figure 3:**
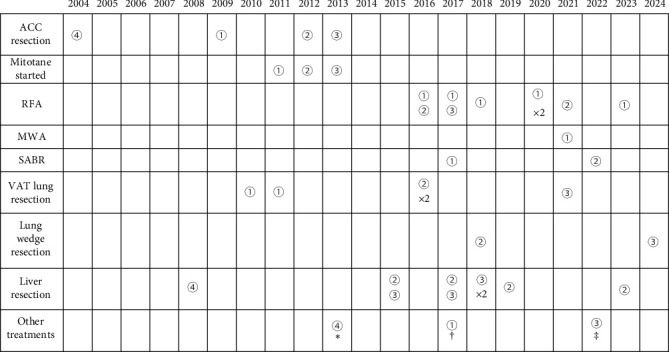
Longitudinal treatments for Case 1, Case 2, Case 3 and Case 4. ①: Case 1; ②: Case 2; ③: Case 3; ④: Case 4. *⁣*^*∗*^Surgical excision of local recurrence, ^†^Surgical removal of transverse colon metastasis through colonoscopy, ^‡^Selective excision of retroperitoneal abdominal lymph nodes; ACC, adrenocortical carcinoma; RFA, radiofrequency ablation; MWA, microwave ablation; SABR, stereotactic ablative radiotherapy; VAT, video-assisted thoracoscopy.

We present four cases of patients with 20-, 15-, 12- and 11-year histories of metastatic ACC, treated with repeated surgeries, long-term mitotane therapy and multiple locally mini-invasive tumoral ablations, without EDP or other chemotherapy schemes (Table 1, Figure 3). Within our records, which included 167 patients with ACC from 2004 to 2024 diagnosed at different stages, these four patients are the only that received prolonged mitotane administration without EDP (Figure 1), reflecting how unique they are. Our four cases well illustrate that no single clinical or biological marker reliably predicts aggressiveness: for example, Case 2 had a high Ki-67 (37%, adverse) but favourable ENSAT Stage II and R0 resection, whereas Cases 1, 3 and 4 showed low Ki-67 yet had either ENSAT Stage III disease or unknown resection status (both adverse) [25]. To our knowledge, this is the first case series with exhaustive documentation of mitotane plasma levels maintained above 14 mg/L for most of the time, not provided by other reports about metastatic ACC long-term survivors. These cases illustrate the complexity of managing patients with metastatic ACC, emphasising the need for a multidisciplinary approach to assure the best possible care and simultaneously optimise disease control and quality of life. This case series also highlights the importance of continuous clinical, biochemical and radiological surveillance to allow a timely and comprehensive intervention. In fact, the regular close monitoring allowed early detection of disease recurrences and enabled prompt MDT review and discussion, which is known to positively affect overall and PFS in patients with metastatic ACC [28]. Within the MDT, a systemic chemotherapy approach with EDP + mitotane was discussed several times for Case 1, Case 2 and Case 3, although it was never administered in consideration of the slow disease progression and location of tumour manifestations (approachable by surgery or other LT), as well as the patient's preference. Hence, our MDT opted for a long-term mitotane therapy associated with LTs and resurgeries. Although surgical excisions, RFA, SABR and microwave ablation have indeed contributed to prolong the survival of the patients (a summary of treatments is in Figure 3 and Table 1), we can speculate that mitotane played an additional role in slowing the progression of the disease, allowing a tailored and local approach at each recurrence. One further possible explanation, though limited to Case 2, could be the lower Ki-67 index observed in three liver metastases (16%, 25% and 30%) compared with the primitive tumour Ki-67 (37%). However, this difference alone is unlikely to fully account for the overall clinical picture.

**Figure 4:**
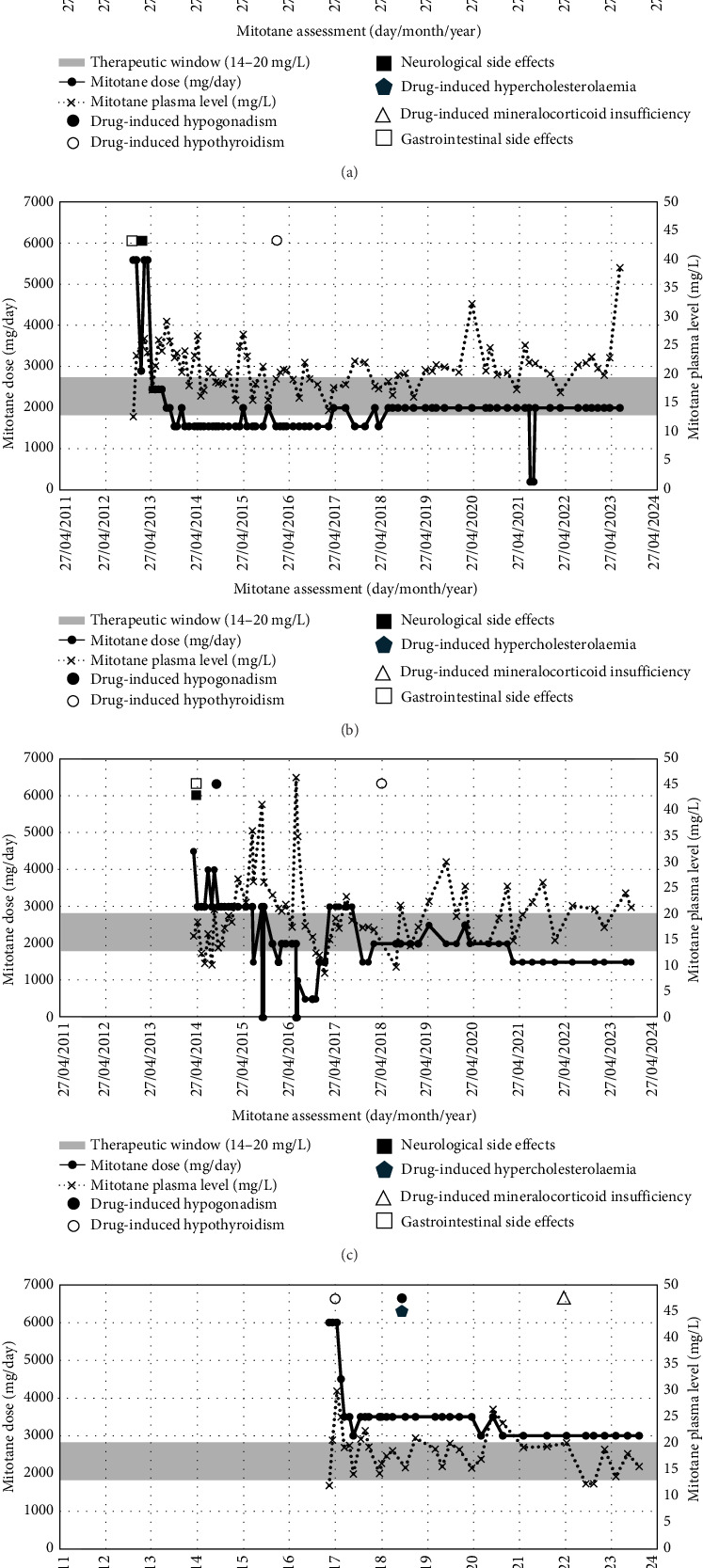
Mitotane plasma level, mitotane dose and side effect events for Case 1 (a), Case 2 (b), Case 3 (c) and Case 4 (d). 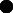 Drug-induced hypogonadism; 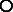 drug-induced hypothyroidism; 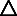 drug-induced mineralocorticoid insufficiency; 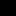 neurological side effects; 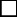 gastrointestinal side effects; 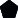 drug induce hypercholesterolaemia (a) Case 1; (b) Case 2; (c) Case 3; (d) Case 4.

In the literature, there are few reports of patients (~30 worldwide) with metastatic ACC and 10 or more years of survival [13–23]. Female sex, age > 40 years and left side of primary tumour are overall the most represented features in this cohort of patients (Table 2). However, these features are described as common characteristics of ACC; hence, it is not possible to indicate them as predictors of long-term survival in patients with metastatic ACC [29]. Even therapies and treatments administered to this cohort are strongly heterogeneous, including mitotane, EDP and other chemotherapy schemes, repeated surgeries and LTs [13–23]. The heterogeneity in terms of treatment approaches might be due to the more recent development of new mini-invasive treatments [2, 3, 13–23, 30]. Interestingly, among these patients, only few were reported receiving mitotane for more than 5 or 10 years, while the mean mitotane treatment duration of our patients is 10.8 ± 2.2 years (±standard deviation) (Table 1). Two patients reported by Ilias et al. [15] in 2001 received mitotane for 14 and 16 years, although no description of mitotane concentrations was provided. More cases of long-term mitotane administration were reported by Terzolo et al. [7] in 2007. Hereby, within the mitotane treatment group (median duration 29 months, range: 6–164 months), 21 patients received mitotane for 4 years or more, but no further details were given [7]. Differently from the patients reported by Ilias and Terzolo, we used the high-saturation dose regimen first described in 2013, leading to a quick increase in mitotane plasma levels even above 20 mg/L within the first 3 months, which has been proved to positively impact the PFS [31]. In the following years, we provided continuous support and management of side effects, while making continuous adjustments to mitotane daily dose according to the mitotane plasma levels, that frequently fluctuated above the toxicity limit for most of the time (especially for Cases 1–3, Figure 4 and Table 1). This was possible thanks to a patient-centred plan led by a specialist nurse that enabled regular clinical assessments and supported self-management of mitotane-induced adrenal insufficiency through ongoing education and monitoring [32, 33]. This model is supported by successful outcomes in the management of other cancers, where specialist nurses have contributed not only to service coordination and psychological support but also to improved overall survival [34, 35].

Our findings may support the hypothesis that prolonged mitotane therapy may be safely and effectively maintained in selected patients with LTB metastatic ACC, even at plasma concentrations exceeding 20 mg/L and for durations longer than 5 years. It should also be considered that our patients spent most of the treatment period within the mitotane therapeutic range, a pattern that may also have contributed to prolonged disease control, as suggested by Puglisi et al. [27]. Although, it is worth to mention that whenever applicable, we arbitrarily reduced mitotane dose when its plasma levels surpassed 35 mg/L. Future research is needed to confirm our findings, which could support the long-term use of the mitotane and LT in similar cases for which current guidelines do not provide clear indications [2, 3].

Our study presents some major limitations, such as the lack of quality-of-life systematic evaluation. The latter has been addressed by few articles on and highlighted the complexity of assessing quality of life in patients with ACC, as it involves psychological distress caused by both oncological disease burden and hormonal insufficiency or excess [36–38]. Nevertheless, continuous support was offered to our patients and their primary care physicians by means of phone and email contacts to cope with mitotane-related side effects and assure compliance with replacement medications and other treatments. This established pathway allowed patients and their families to engage with our clinical care, in which endocrine nurses played a pivotal role in addressing patients' concerns, not only by supporting the management of physical symptoms but also by attending to emotional, mental and psychological wellbeing through coaching and signposting to patient associations and support groups.

## 8. Conclusion

Metastatic ACC is a challenging malignancy that requires continuous monitoring and timely treatment that only a multidisciplinary approach can provide. Few similar cases of metastatic ACC long survivors have been reported worldwide, with no shared characteristic that can be isolated for outcome prediction, while fewer reported mitotane use longer than 5 years. With this case series, we highlight the successful integration of multiple surgical resections, localised treatments and long-lasting mitotane therapy with highly specialised nurse support to achieve long-term disease control in LTB metastatic ACC. Moreover, we provided real-world evidence supporting the safety of mitotane administration for more than 5 or even 10 years. Long-term treatment with mitotane was associated with relatively good quality of life and manageable drug-related side effects.

## Figures and Tables

**Table 1:** Summary of case presentation, mitotane and recurrences management.

Features	Case 1	Case 2	Case 3	Case 4
Presentation at diagnosis				
Sex	Male	Female	Male	Male
Age	49	52	34	39
Previous smoke habit	Yes	No	No	No
Previous alcohol habit	Yes	No	No	No
Symptoms	Yes (mass effect)	Yes (weight gain and virilization)	Yes (mass effect)	Yes (mass effect)
Hormonal secretion	Unknown (likely inactive)	Glucocorticoid and androgen excess	Glucocorticoid and androgen excess	Inactive
Clinical examination	Negative	Cushing syndrome	Cushing syndrome	Negative
Weiss score	5	9	8	Unknown
Ki 67 index (%)	<1	37	4.4	8–10
ENSAT Stage	3	2	3	2
Resection status	Rx	R0	R0	RX
S-GRAS score	3	5	2	3
Mitotane management				
Average mitotane plasma level (mg/L ± SD)	21.10 ± 5.46	21.88 ± 4.47	20.06 ± 7.11	18.42 ± 3.98
Average mitotane dosage (g/day ± SD)	2.8 ± 1.1	2.0 ± 9.8	2.2 ± 8.5	3.6 ± 8.1
Years of mitotane	12.9	11.8	10.6	7.7
Time below target range (<14 mg/L) (%)	9.7	2.6	15.0	12.1
Time in target range (14–20 mg/L) (%)	41.7	65.4	41.7	27.3
Time above target range (> 20 mg/L) (%)	48.6	32.0	43.3	60.6
Induced hypothyroidism	Yes	Yes	Yes	Yes
Induced hypogonadism	Yes	No	Yes	Yes
Induced mineralocorticoid insufficiency	Yes	No	No	Yes
Induced hypercholesterolaemia	No	No	No	Yes
Neurologic side effects requiring dose adjustment	Yes	Yes	No	No
Gastrointestinal side effects requiring dose adjustment	No	Yes	Yes	No
Recurrences management				
Time to first recurrence (months)	8	44	20	~48
SABR treatments	1	1	0	0
MWA treatments	1	0	0	0
RFA treatments	6	1	1	0
Liver resection (NAR or HH)	0	4	5	1
Lung resection (VAT or wedge)	2	4	2	0
Other^a^	1	0	1	1
Disease status at last follow-up	Free	Free	Free	Free
Years of follow up	15	12	11	20

*Note:* Rx: resection status unknown; R0: tumour fully excised.

Abbreviations: ENSAT, European Network for the Study on Adrenal Tumours; HH, hemi-hepatectomy; MWA, microwave ablation; NAR, non-anatomical resection; RFA, radiofrequency ablation; S-GRAS, Stage, Grade, Resection status, Age, Symptoms; SABR, stereotactic ablative radiotherapy; SD, standard deviation; VAT, video-assisted thoracoscopy.

^a^Case 1: Surgical removal of transverse colon metastasis through colonoscopy; Case 2: None; Case 3: Selective excision of retroperitoneal abdominal lymph nodes; Case 4: Surgical excision of local recurrence.

**Table 2:** Patients with metastatic ACC and survival longer than 10 years.

Author (publication year)	Age (years)	Sex	OS (years)	Side	Size (cm)	Hormones	Symptoms	ENSAT stage
Aalderen et al. [20] (1992)	NA	NA	22	NA	NA	NA	NA	NA

Sakamoto et al. [19] (1995)	40	F	18	L	NA	Inactive	NA	3

Ilias et al. [15] (2001)	39	F	14	L	9	Cortisol	Cushing	2
	24	F	16	L	19	Cortisol	Hypertension, amenorrhoea	2

Orlando et al. [14] (2003)	58	M	15	NA	NA	Inactive	NA	NA

Meyer et al. [13] (2007)	34	F	32	L	17	Inactive	Mass related	2

Hamanaka et al. [22] (2008)	40	F	16	L	14	Inactive, AFP^a^	NA	NA

Hermsen et al. [16] (2008)	53	F	12	L	10	Cortisol	Cushing	2
	38	F	17	L	14	Inactive	Mass related	4
	34	F	18	R	12	Cortisol	Cushing	2
	49	F	18	L	7	Inactive	Mass related	2
	31	F	25	R	9	Aldosterone	Hypertension	2
	22	F	28	L	8	Cortisol	Cushing	2

Tran et al. [17] (2016)	33	F	10	NA	20	NA	NA	3
	32	F	11	NA	NA	Androgens	Virilization	NA
	49	F	12	NA	13	Inactive	NA	2
	42	F	12	NA	9	Inactive	NA	2
	55	M	12	NA	10.5	Inactive	NA	4
	73	F	14	NA	4.5	Inactive	NA	1
	57	M	11	L	4.5	NA	Mass related	1

Kostiainen et al. [23] (2019)^b^	NA	NA	>10	NA	NA	NA	NA	NA

Mauda-Havakuk et al. [21] (2021)^c^	NA	NA	24	NA	NA	NA	NA	NA

Current study	34	M	11	L	21	Cortisol	Cushing	3
	52	F	12	L	22	Cortisol	Cushing	2
	49	M	15	L	21	Likely inactive	Mass related	3
	39	M	20	R	*NA*	Inactive	Mass related	2

*Note:* ENSAT stage, where not specified, was calculated according to reported mass diameter and tumour stage.

Abbreviations: AFP, alpha-fetoprotein; ENSAT, European Network for the Study on Adrenal Tumours; F, female; L, left; M, male; NA, not available; OS, overall survival; R, right.

^a^Metastasis secreting AFP.

^b^One single patient undergone multiple treatment for metastatic ACC with survival >10 years from a cohort of 43 adult patients with ACC.

^c^Patient with longest survival among 10 patients reported with metastatic ACC and survival >10 years.

## Data Availability

The data obtained for this article were collected from the electronic archives of Queen Elizabeth Hospital Birmingham. The longitudinal trends for mitotane plasma levels and daily dose displayed in Figure 3a,b,c,d were retrospectively retrieved from Lysosafe Service (https://lysosafe.clinfile.com). The data that support the findings of this study are available on request from the corresponding author. The data are not publicly available due to privacy or ethical restrictions.

## Nomenclature

ACC: Adrenocortical carcinoma
CT: Computerised tomography
EDP: Etoposide, cisplatin, doxorubicin
ENSAT: European Network for Study of Adrenal Tumour
FDG-PET-CT: 18-fluorodeoxyglucose positron emission tomography-CT
LS-AMRI: Liver-specific abdomen magnetic resonance imaging
LT: Local therapy
LTB: Low tumour burden
MDT: Multidisciplinary team
NAR: Non-anatomical resection
PFS: Progression Free Survival
RFA: Radiofrequency ablation
Rx: Resection status unknown
SABR: Stereotactic ablative radiotherapy
S-GRAS: Stage, Grade, Resection status, Age, Symptoms
TAP: Thorax–abdomen–pelvis
TTR: Time in target range
VAT: Video-assisted thoracoscopy.
